# Side effects and the need for secrecy: characterising discontinuation of modern contraception and its causes in Ethiopia using mixed methods

**DOI:** 10.1186/s40834-017-0052-7

**Published:** 2017-10-19

**Authors:** Alexandra Alvergne, Rose Stevens, Eshetu Gurmu

**Affiliations:** 1 School of Anthropology & Museum Ethnography, University of Oxford, 51/53 Banbury road, Oxford, OX2 6PE UK; 2 Center for Population Studies and Institute of Development and Policy Research, Addis Ababa University, Addis Ababa, Ethiopia

**Keywords:** Ethiopia, Family planning, Contraceptive discontinuation, Side-effects, Multilevel multiprocess modelling, Semi-structured interviews, Reproductive ecology, Unmet needs, Mixed methods, Education

## Abstract

**Background:**

Contraceptive discontinuation is a major barrier to reducing global unmet needs for family planning, but the reasons why women discontinue contraception are poorly understood. Here we use data from Ethiopia to investigate (i) the magnitude of contraceptive discontinuation in 2005–2011, (ii) how the risk of discontinuation varies with method type and education level and (iii) the barriers to continuation. Our main hypothesis is that contraceptive discontinuation is driven by the experience of physiological side-effects associated with the use of hormonal contraception, rather than a lack of formal education.

**Methods:**

We used a mixed methods explanatory sequential design to explain the quantitative results in more details through the qualitative data. First, we analysed quantitative data from the 2011 Ethiopian Demographic and Health Survey to study patterns of contraceptive discontinuation and method choice using multilevel multiprocess models. Second, we conducted semi-structured interviews and focus group discussions in the 3 most populated regions of Ethiopia with individuals of reproductive age and health professionals.

**Results:**

The analysis of EDHS data shows that the rate of discontinuation has not reduced in the period 2005–2011 and remains high. Discontinuation mainly takes the form of abandonment, and is a function of method type, age and wealth but not of educational level. Interviews with women and health professionals reveal that the experience of debilitating physiological side effects, the need for secrecy and poverty are important barriers to continuation.

**Conclusions:**

Our findings together suggest that physiological and social side-effects of contraceptive use, not a lack of formal education, are the root causes of contraceptive abandonment in Ethiopia.

## Background

### Contraceptive discontinuation as a major determinant of unmet needs

Unwanted fertility leads to increased rates of abortion and maternal mortality, and acts as a major barrier to improving individual health, gender equity, family wellbeing and national development [1–4]. Thus, tackling unmet need for contraception, i.e. the proportion of women wishing to limit or postpone child birth but who are not using contraception [5], has recently been highlighted as a key global health issue [6]. In low-resource countries, 1 in 4 women has an unmet need for family planning and 79% of unintended pregnancies occur among these women [2]. Yet, a main obstacle to reduce unwanted fertility is the discontinuation of modern contraception [7–9], a currently underappreciated proportion of unmet need. On average, over a third of women who start using a modern contraceptive method stop using it within the first year, and over a half stop before 2 years [9]. Following Jain’s claim that programs would do better to concentrate on retaining existing users rather than focus on recruiting new clients [10, 11], recent reports have suggested that tackling contraceptive discontinuation is central to achieving better demographic outcomes [7, 8]. To date, however, there is little valid literature on either the reasons for discontinuation [12] or the programmatic interventions explicitly designed to reduce discontinuation [9].

### The reasons for contraceptive discontinuation

Why women discontinue contraception is multi-faceted, but several reviews of DHS data have demonstrated that side effects and health concerns associated with the use of hormonal contraceptives are major reasons for discontinuation [7–9, 11, 13]. In a review of oral contraceptive (OC) discontinuation in 19 developing countries, Ali & Cleland [7] found that the dominant reason for terminating OC use within 12 months is dissatisfaction with the method, predominantly side-effects and health concerns. These account for a median of 28% of all reasons for discontinuation, reaching as high as 40% in Bolivia [13]. Yet, most current studies are based on DHS data, which only records one general reason for discontinuation and one for non-use. Answers are then often grouped into large ambiguous categories such as ‘health concerns’ or ‘opposition to use’ making it difficult to understand the nuances behind the reasons [14]. There have been several qualitative studies on attitudes towards contraceptive use that cite health concerns as a barrier to contraceptive use and continuation [15–18], but, similarly to DHS data, the distinction between unsubstantiated fears and the experience of side effects is not systematically articulated. This is problematic because it can lead to the assumption that concerns about side-effects are grounded in myths and misconceptions that can be alleviated by counselling and education [5, 8, 9], disregarding the possibility that side-effects may be intolerable [19].

### Hormonal contraceptives and physiological side-effects

Physiological side-effects associated with the use of hormonal contraception have been hypothesized to result from a mismatch between a woman’s endogenous hormonal levels and the dosage of contraceptives [13, 20]. The dosage of synthetic ovarian steroids in hormonal contraceptives is balanced to be high enough to prevent ovulation, but not too high so as not to lead to side-effects (e.g. nausea, vomiting, and headache) [21]. This balance is likely to be offset in the case of many women living in the developing world because contraceptives are generally trialled on Western, industrialized and non-seasonal populations, and thus contain high levels of ovarian steroids. In addition, there is widespread evidence that female reproductive functioning is highly variable between populations of different subsistence modes [20, 22, 23], and within populations, between seasons, socio-economic classes and migration status [24, 25]. This suggests that health concerns might be rooted in the experience of side-effects, independently of both education level and knowledge of reproductive health.

### Contraceptive discontinuation in Ethiopia

In Ethiopia, the focus of this paper, contraceptive prevalence has increased nine-fold from 1990 to 2011 and to date, it continues to rise. The increase in the number of contraceptive users speaks, to some extent, to the success of the Health Extension Program launched by the Federal Ministry of Health (FMOH) of Ethiopia in 2003 to reach the under-served in rural areas [26, 27]. However, based on the analysis of data from Demographic and Health Surveys (DHS) from 25 countries including DHS data collected in Ethiopia in 2005, it was found that both the level of unmet need for contraception (33.8%) and contraceptive discontinuation due to method-related dissatisfaction were the highest in Ethiopia [8]. Although the picture has somewhat improved, with unmet needs down to 22.3% in 2016, there is scope for further decreasing unmet needs for contraception. A promising avenue might be to make the tackling of contraceptive discontinuation a priority. Since 2005, however, there has been no in-depth investigation of the magnitude and causes of contraceptive discontinuation in Ethiopia.

### Aim of the study

This paper seeks to characterize and improve understanding of the determinants of contraceptive discontinuation in Ethiopia, using both quantitative and qualitative data. Specifically, we aimed to investigate (i) the magnitude of contraceptive discontinuation at the country level in 2011, and how it compares to the situation observed in 2005, (ii) the roles of method type and education level in modulating the risk of contraceptive discontinuation and (iii) the barriers to contraceptive continuation. Our main hypothesis is that contraceptive discontinuation is driven by the experience of physiological side-effects associated with the use of hormonal contraception, rather than a lack of education.

## Methods

### Study design and population

First, we conducted a statistical analysis of a nationally representative sample of women of reproductive age using the Ethiopia 2011 DHS Survey, focusing on unsterilized women who have ever used modern contraception in the preceding 5 years of the survey. Because method choice and discontinuation are not independent, i.e. women who might want to discontinue are more likely to choose short over long acting methods [11], we used a multilevel and multiprocess model [28] to avoid overestimating the discontinuation of short-acting methods. Second, given the Ethiopian 2011 DHS data does not inform on the reasons for discontinuation, a qualitative study on the experience of contraceptive use was run to supplement the quantitative analysis.

### Analysis of the Ethiopian 2011 demographic and health survey (EDHS)

The ethical procedure associated with DHS data collection can be found on the DHS website [29]. The contraceptive histories are collected using a “calendar” that records monthly contraceptive status during the 5 years preceding the survey. The 2011 EDHS dataset includes 16,515 women, married and unmarried, aged 15–49, among which 30.56% have ever used a contraceptive method (29.15% have ever used a modern contraceptive method and 17.93% are currently using a method). Following previous analyses [28], we distinguished 3 types of discontinuation: failure (unintentional) as opposed to abandonment and switch (intentional). Failure corresponds to a situation in which a woman reports becoming pregnant while using a given method (so it includes failure of the method itself and failure to use it). Abandonment corresponds to the absence of use for more than a month and may lead to an immediate risk of unwanted pregnancy. Switch corresponds to a switch from one method to another within a month and as defined here, does not lead to periods during which a woman is unprotected. We focused on modern methods and distinguished 4 different types (1) oral contraceptives (OC), (2) injectables, (3) IUD and implants (long term methods which require health workers to remove it) and (4) condoms. Episodes of contraceptive use contributed by sterilised women (1.4% in 2011) were excluded, as sterilisation is non-reversible. When discussing condoms, we are referring to male as opposed to female condoms.

#### Modelling contraceptive discontinuation and method choice: An overview

To analyse the risk of contraceptive discontinuation over time, the data have been converted into a period-person dataset, considering the unit of analysis to be an episode of contraceptive use. An episode is defined as a continuous period of use of a specific method. Once an individual has experienced one type of discontinuation, the individual is removed from the observations; the survival time for the competing risks are latent and we only observe *T* = min (*T*(failure), *T*(switch), *T*(abandon)) or the censoring time if no discontinuation has occurred [28]. We concentrate on women who have ever-used a modern contraceptive method (*N* = 4814), excluding from this sample the women who only contributed episodes of contraceptive use that started before the calendar period (*N* = 111), as the start dates of those episodes is not known. We also excluded episodes of contraceptive use that referred to sterilization (*N* = 246). The analysis thus includes data on 7022 episodes of use contributed by 4703 ever-users. Within those episodes of contraceptive use, 3965 events of discontinuation were observed (56.5%). In the case of discontinuation events, 64.6% correspond to abandonment; 15.9% correspond to failure and 19.5% correspond to switching.

Following Steele & Curtis [28], the analysis proceeds in 2 steps using the aML statistical software (http://www.applied-ml.com). First, as there are 3 types of discontinuation, we used a competing risk hazard model, with one equation per risk of discontinuation. The model allows for unobserved heterogeneity between women (i.e. random effects) that may influence the duration of each episode. To investigate if the risks are correlated (e.g. the risk of failure is not independent from the risk of abandonment if a woman wants to avoid pregnancy), the random effects must be correlated across the different types of discontinuation; and thus the 3 equations must be estimated simultaneously. Second, because some women who are more likely to discontinue are more likely to use short-acting methods, the analysis must consider that method choice is a potential endogenous variable. To address this issue, we use a multilevel multiprocess model that simultaneously models the processes of contraceptive choice and contraceptive discontinuation. In all analyses, our variables of interest were method type and a woman’s educational level. We all also included covariates that have been previously found to relate to contraceptive discontinuation, i.e. household socio-economic status, area of residence, religion and the age of the women at the start of the contraceptive episode [30]. In the data, 87% of women are living with a partner in 2011. We did not include current marital status in the analysis because (i) it is not known whether or not it has changed during the 5 years’ calendar period and (ii) although marital status has usually been taken as a proxy for sexual activity and exposure to pregnancy [31], it might not be good indicator. Among married women, postpartum abstinence of up to 1 or 2 years is common [32], which might decrease rather than increase the risk of unwanted pregnancy. Among unmarried women, sexual activity is complex as this group includes both never and formerly married women who might differ in their behaviour and/or their report of sexual activity. Overall, there is considerable diversity of levels of sexual activity amongst both married and unmarried women [33]. Each step of the analysis is further detailed below.

#### Standard multilevel hazards models for competing risks

First, we modelled discontinuation using a competing risk hazard model. We built one model per type of discontinuation step by step: (i) estimation of a simple Gompertz hazard model, without covariates; (ii) adding heterogeneity by incorporating residuals following a univariate normal distribution; (estimating the unobserved woman-level effect). The inclusion of random effects enables to account for unobserved woman-level characteristics that influence the hazard of discontinuation at each month of a given episode and for each episode. The 3 types of discontinuation were modelled jointly (3 equations) to allow for the possibility that random effects are correlated across equations. Then, we ran the 3 models simultaneously, first with uncorrelated random effects (e.g. assuming that the risks do not compete) and then with correlated random effects (assuming that there are competing risks). We found that those effects were not correlated (Table 1) thus single hazard models (one per type of discontinuation) were subsequently modelled.

**Table 1:** Estimated standard deviations and pairwise correlations for woman-level random effects from the multilevel models (EDHS 2011)

	Type of discontinuation		
Random effects	Failure	Abandon	Switch
Failure	1.14***(0.18)		
Abandon	0.07(0.25)	1.08***(0.08)	
Switch	0.52(0.36)	0.27(0.24)	1.02***(0.18)

The correlations between random effects are not significant therefore they are constrained to 0 in the following analyses. It means that hazards models can be modelled independently. Bold depicts significance: *P < 0.05, **P < 0.01, ***P < 0.001

#### Method choice

We used a multinomial probit model. Multinomial probit models are appropriate when the outcome of interest takes on a limited number of values that are not ordered. As compared to a logit model, it is particularly useful as it relaxes the hypothesis of independence of alternatives (IAA). Indeed, whether or not a woman will choose to use oral contraceptives might depend on the availability of condoms (another short-acting method). We thus ran a multinomial probit model with 4 possible alternatives (1) OC; (2) intra-uterine device (IUD) and implants; (3) injectables; (4) condoms. The way dependence between alternatives is considered is by correlating the residuals across the equations. Other methods of contraception are used in Ethiopia but we were only able to consider 4 choices due to the limitations of the aML software.

#### Extension: Multilevel multiprocess (allows for endogeneity)

Discontinuation and method choice were modelled jointly to allow for the fact that some women who are more likely to discontinue are also more likely to use short-acting contraceptives (i.e. the endogeneity of method choice). In effect, women level random effects are correlated between the discontinuation equations and the method choice equation [28]. We only considered 3 choices (OC, injectables and condoms) due to software limitations.

### Interviews and focus group discussions (FGD)

Ethical approval for the collection of qualitative data has been sought from and granted by the Institute of Development and Policy Research of Addis Ababa University (Ref IDPR/LT-04209/16). Informed consent was obtained by all participants verbally before interviews and FGDs began. A written page to obtain informed consent was read out for each of the participants and the necessary explanation was given to each of the potential participants depending on the further clarification and additional information they required.

#### Study sites, recruitment of participants and description of the sample

Interviews and focus group discussions (FGDs) were conducted in 2013 in three health centres in the three most populated regions of the country (the rural Arsi Administrative Zone of Oromia Region, the Sidama Administrative Zone of SNNP Region and the North Shewa Administrative Zone of Amhara Region). These health centres were selected on the basis of 3 different regions to give a variety of opinion from different traditionally bounded areas. The specific health centres were chosen for convenience of location and availability for visit by the researchers. Interviewees were approached at health facilities whilst visiting family planning (FP) clinics through the help of FP service providers or at their residence through the help of local persons serving as interview or FGD facilitators. Service providers were interviewed in order to understand their perceptions for the main reasons that their clients continue or discontinue contraception as they have direct experience with many women who seek advice regarding contraception. There were no direct refusals to participate. However, some individuals were willing to take part in the study but were unable to, due to their busy schedule or inconvenience of time and location of the interview or FGD.

Qualitative data was collected using 9 in-depth interviews and 3 FGDs in total among ever-married women of reproductive age (18–49) that had ever used contraception and 6 key informant interviews among service providers (i.e. nurses and midwives) working in health facilities and health posts. These were distributed as 5 interviews per site (3 women and 2 service providers) and 1 FGD with women per site with 8, 10 and 7 women in Arsi, Sidama and Shewa sites respectively. Different women were approached for interviews and FGDs and therefore a total of 34 women and 6 health care providers were spoken to. In all cases, only ever-married women were included as discussing sensitive issues related to sexual behaviour before marriage is not well accepted in such traditional societies of Ethiopia and only women over 18 were spoken to as that is the legal age of consent in Ethiopia.

In recruiting participants, inclusion criteria were selecting individuals of different socioeconomic classes: poor, middle and better off individuals among residents in the community and of different levels of educational attainment: no-education, primary (1–8 grades) and secondary and above (grade 9 and higher achievers). The sampling was designed to include a range of different socio-economic classes and residential locations to give a broad insight into some of the factors that might explain contraceptive discontinuation in Ethiopia.

#### Data collection procedure and instruments

The individual interviews and FGDs were conducted within the same visit at each of the regions with the aim to capture personal experiences and opinion through in depth interviews and consensus or divergent views in the FGDs. Interviews were conducted beforehand whist FGD participants and timings were arranged. The interviews took place during an appointment at health facilities or at their residence depending on the preference of the interviewee. FGDs were done on school compounds or public places depending on availability and suitability of meeting places. The responses obtained from both the in-depth interviews and the FGDs were thematically analysed together to incorporate views expressed in private and as a group in the results.

An experienced female research assistant conducted the interviews and FGDs, among women using local languages and lasting on average one and two hours, respectively. The interviews among service providers were conducted by the female research assistant or the last author, a male Ethiopian citizen working and living in the country, depending on location. The 2 service providers at each location consisted of 1 health extension worker (female) and 1 zone health office worker/supervisor (male or female) per location.

The interviews and FGD aimed at providing a deeper understanding of the major issues of enquiry generated from the statistical results such as (1) Why do women discontinue contraceptive use in Ethiopia? (2) What sociocultural factors affect contraceptive discontinuation? (3) Why are short-acting hormonal methods both the most used and most discontinued? These questions, arising from the gaps in understanding left after the completion of the statistical analysis, informed the creation of the interview and FGD guides (see below). As this is the first study of its kind looking into contraceptive discontinuation in Ethiopia we formulated our own guides based upon these questions.

The questions asked in female interviews were as follows:

1. *How often do you use contraception?*
  - *What is the longest time you have used the same contraceptive continuously? Why?*
2. *Why do you use family planning methods?*
  - *Could you tell me some of your specific reasons?*
3. *Where do you get the contraceptive supplies?*
  - *Is it at your vicinity or at a distant location?*
  - *Why do you prefer to go there?*
4. *What type of contraception are you using?*
  - *Could you tell me why you have chosen such a method?*
  - *Was it the type of contraceptive you have used from the very beginning?*
5. *What challenges/problems were you facing in using contraception?*
  - *Could you explain it to me further?*
  - *How long have you faced such a problem?*
  - *What measures have you taken to overcome such a problem?*

Focus group questions were similar but asked about the wider community’s contraceptive use and attitudes:

1. *How often do people in your community use contraception?*
2. *Why do people in your community use family planning methods?*
  - *Could you tell us some of their specific reasons?*
3. *Where do people in your community get contraceptive supplies?*
  - *Is it at their vicinity or at distant location?*
  - *Why do you think they prefer to go to those places?*
4. *What type of contraception do people in your community often use?*
  - *Could you tell us why they would choose such a method(s)?*
  - *Is it the type of contraceptive they often take up from the very beginning?*
5. *What challenges/problems do women of reproductive age face in using contraceptives?*
  - *Could you explain them further?*
  - *What measures do they often take to overcome such a problem(s)?*

Service providers were asked the same questions as in the FGDs in their interviews with the addition of one extra question: What are the views of clients in using family planning methods? These interviews were conducted at respective workplaces of the service providers. All interviews and FGDs were tape recorded and transcribed in the language used for the interview and discussion. The summary of the major findings of the interviews and FGDs were then translated into English for inclusion in the paper. The findings were analysed thematically by grouping them into the major arising themes to improve understanding of the barriers to continuation of contraception.

## Results

### Quantitative findings

#### Trends of contraceptive discontinuation between 2000 and 2011

**Fig. 1:**
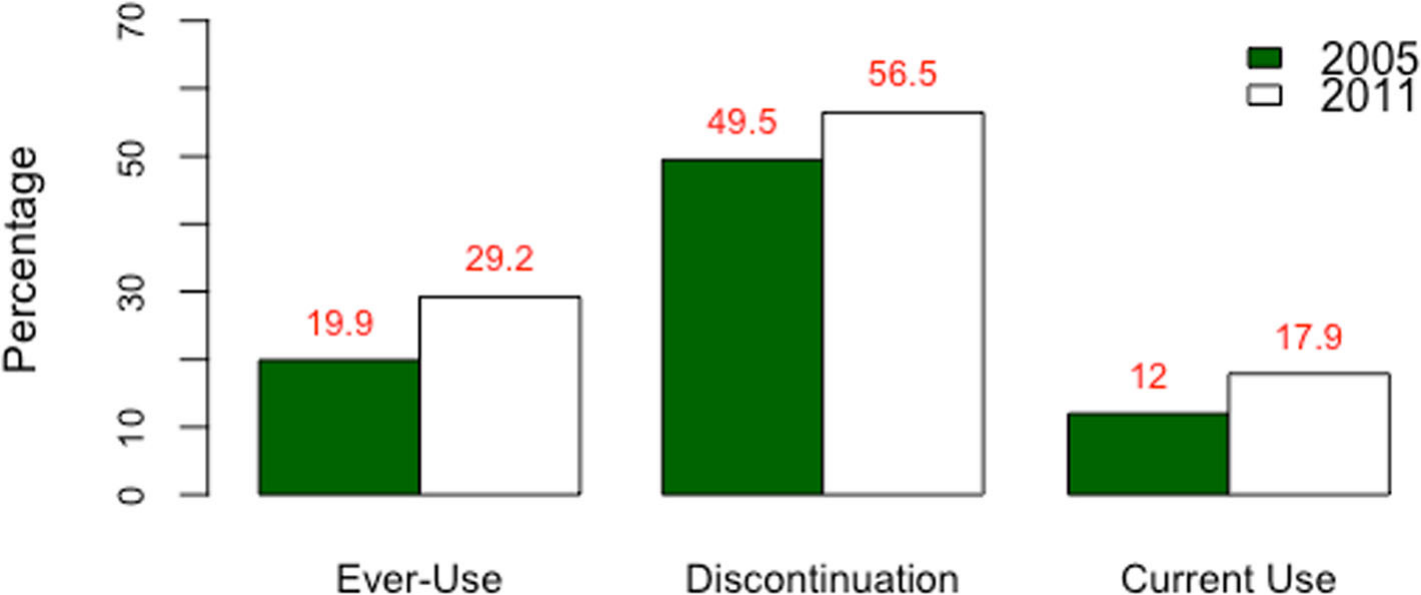
Trends in contraceptive adoption, discontinuation and use among Ethiopian women, 2005–2011. This figure is based on the descriptive statistics of the data collected during the 2005 and 2011 Ethiopian demographic and health surveys. The percentage of ever-users of modern contraception has increased by ca. 10% over the period investigated. The percentage of contraceptive discontinuation, which includes failure, switch and abandonment, has not reduced. The percentage of women using contraception at the time of the survey, i.e. contraceptive prevalence, has increased by ca. 5% in the study period

We compared the magnitude of contraceptive discontinuation between 2000 and 2011, using 5-years calendar data on contraceptive use from both the 2005 and 2011 Ethiopian DHSs. In the period 2006–11, the risk of discontinuation among women who use modern contraceptive methods is high: an episode of contraceptive uptake has a 56.5% risk of terminating, of which 64.6% correspond to abandonment, 15.9% correspond to failure and 19.5% correspond to switching. The overall level of discontinuation is slightly higher than in the period 2000–2005 (analysis of the 2005 EDHS not shown), and the persistence of a high level of discontinuation over the years might partly explain why the increase in contraceptive prevalence does not match the increase in the number of contraceptive ever-users (Fig. 1).

#### The determinants of contraceptive discontinuation

We investigated the hypothesis that contraceptive discontinuation is driven by the type of contraceptive method, but not formal education, among women who have ever-used contraception (see Table 2 for a description of the sample of ever-users). The results show that education has an impact on method choice. Once that effect is controlled for, education level modulates the risk of contraceptive switch, but not that of failure or abandonment. Method type influences all three forms of discontinuation.

**Table 2:** Description of the data

Characteristics	All women		Ever-users of modern contraception	
	*N*	%	*N*	%
Total sample size	16,515		4814	
Age (years)				
< 25	1377	8.3	40	0.8
25–34	6956	42.1	1919	39.9
35+	8182	49.5	2855	59.3
Education				
None	8278	50.3	2177	45.2
Primary	5858	35.5	1760	36.6
Secondary+	2379	14.3	877	18.2
Wealth				
Low	6113	37.0	1106	23.0
Medium	4773	28.9	1469	30.5
High	5629	34.0	2239	46.5
Religion				
Orthodox	6995	42.3	1258	26.1
Muslim	6170	37.3	2715	56.4
Other	3350	20.3	841	17.5
Area				
Rural	11,186	67.7	2786	57.9
Urban	5329	32.2	2028	42.1

The characteristics of the overall 2011 EDHS sample are compared to the subsample of women who have ever used modern contraception. Ever-users tend to be older, more educated, wealthier, more urban and Muslims compared to the overall dataset

##### Method choice

**Fig. 2:**
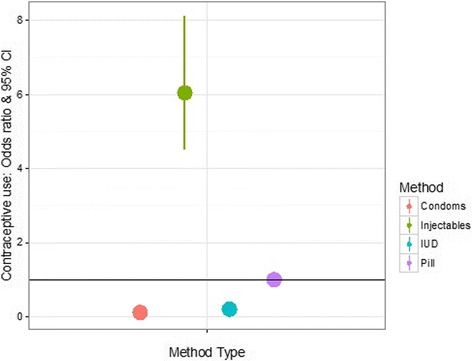
Predicted odd-ratios & 95% confidence intervals for method choice. The reference category (horizontal line) corresponds to the use of oral contraceptives. At the national level, injectables are ca. 6 times more likely to be used than any other short-acting methods. This figure is based on the results of a multinomial probit model using the calendar data collected in the 2011 Ethiopian Demographic and Health Survey

Educated women are more likely to use oral contraceptives and condoms, while less educated women are more likely to use injectables and IUD/Implants (Table 3). However, injectables are the most popular method choice overall and nationally, women are 6 times more likely to use injectables compared to oral contraceptives (OR: 5.99; 95CI [4.48; 8.08]; Fig. 2). The analysis controls for differences in age at the beginning of an episode of contraception, wealth, area, religion and patterns of correlation between methods types.

**Table 3:** Estimated coefficients and standard errors for multinomial probit model on contraceptive method choice (EDHS 2011)

Method choice	IUD/Implants		Injectables		Condoms	
Fixed Effects	Estimate	Std. Err.	Estimate	Std. Err.	Estimate	Std. Err.
Constant	−1.597***	**0.192**	1.799***	**0.150**	−2.176***	**0.400**
Education						
No education (Ref)	0	0	0	0	0	0
Primary	−0.331***	**0.0777**	−0.171***	**0.063**	0.163	0.128
Secondary	−0.403***	**0.0844**	−0.749***	**0.068**	0.434	0.116
Age at start						
< 25 years (Ref)	0	0	0	0	0	0
25–34 years	0.217*	**0.119**	−0.014	0.088	−0.460***	**0.141**
35–49 years	0.560***	**0.119**	−0.349***	**0.089**	−0.891***	**0.147**
Wealth						
Low (Ref)	0	0	0	0	0	0
Medium	0.394***	**0.139**	0.149	0.120	NA	NA
High	0.026	0.142	−0.097	0.117	0.897**	**0.395**
Area						
Urban(Ref)	0	0	0	0	0	0
Rural	0.805***	**0.084**	0.889***	**0.069**	−0.447 ***	**0.137**
Religion						
Orthodox (Ref)	0	0	0	0	0	0
Muslim	−0.006	0.070	−0.183***	**0.058**	−1.232***	**0.131**
Others	0.519	0.484	0.481	0.497	NA	NA
Correlation between women-level random effects across equations						
IUD/Im. & Injectables	−0.097 (0.030) ***					
IUD/Im. & Condoms	−0.302 (0.188)					
Condoms & Inject	−0.077 (0.052)					

The reference category is oral contraceptives. Bold depicts significance: *P < 0.05, **P < 0.01, ***P < 0.001

##### Risk of discontinuation by education level

**Fig. 3:**
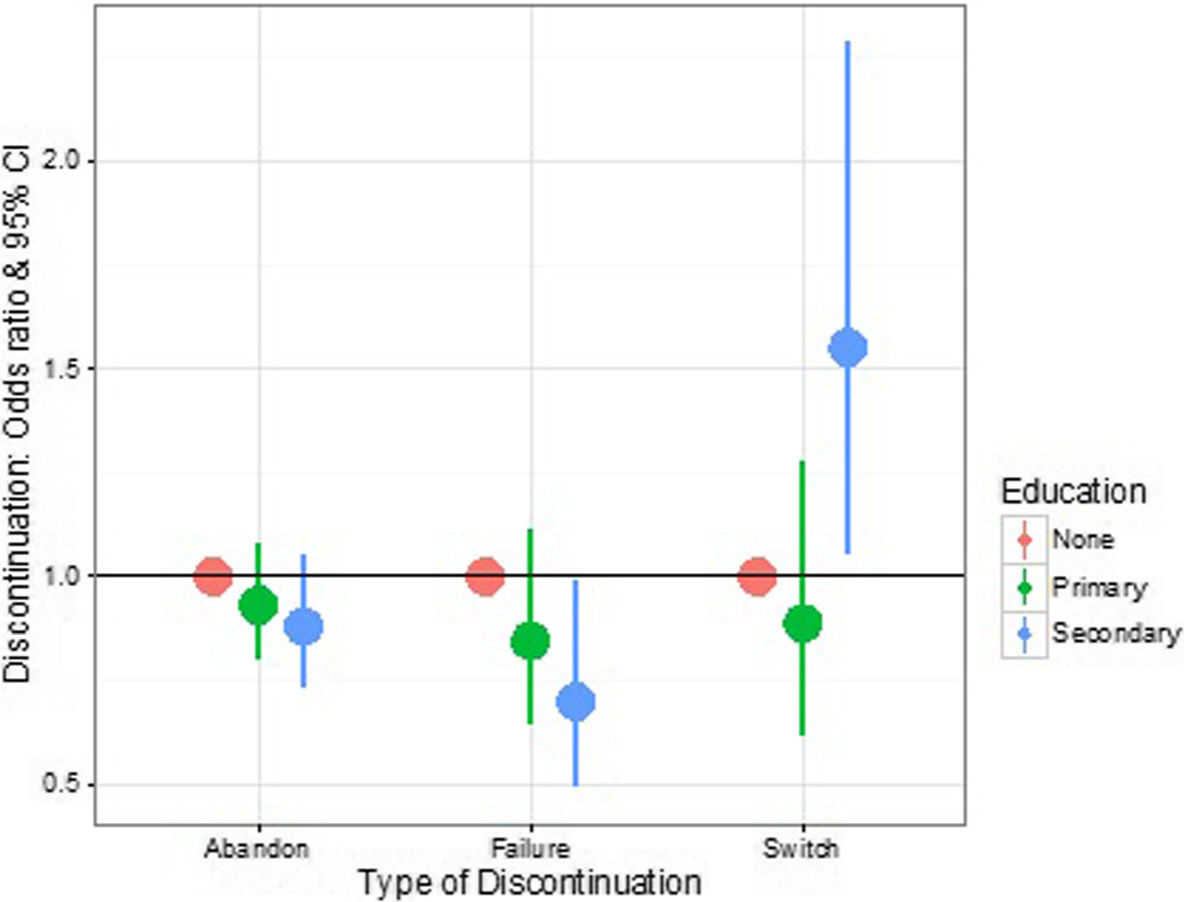
Predicted odd-ratios & 95% confidence intervals for contraceptive discontinuation as a function of a woman’s level of education. The reference category (horizontal line) is no education. The risk for a woman to discontinue contraception because of abandonment (a woman stops using contraception) or failure (a woman becomes pregnant) is independent from her educational level. However, the risk of switching between methods is ca. 55% higher for women with the highest level of education. This figure is based on the results of a multilevel multiprocess model using the calendar data collected in the 2011 Ethiopian Demographic and Health survey

We analysed contraceptive discontinuation as a function of method type and socio-demographic variables, accounting for the effect of socio-demographic variables on the type of method used (multiprocess model). We found that educated women are not more or less at risk of both contraceptive abandonment and failure. However, women with the highest level of education are 55% more at risk to switch methods as compared with the uneducated (OR = 1.55; 95CI [1.05; 2.29]; Fig. 3).

##### Risk of discontinuation by method type

**Fig. 4:**
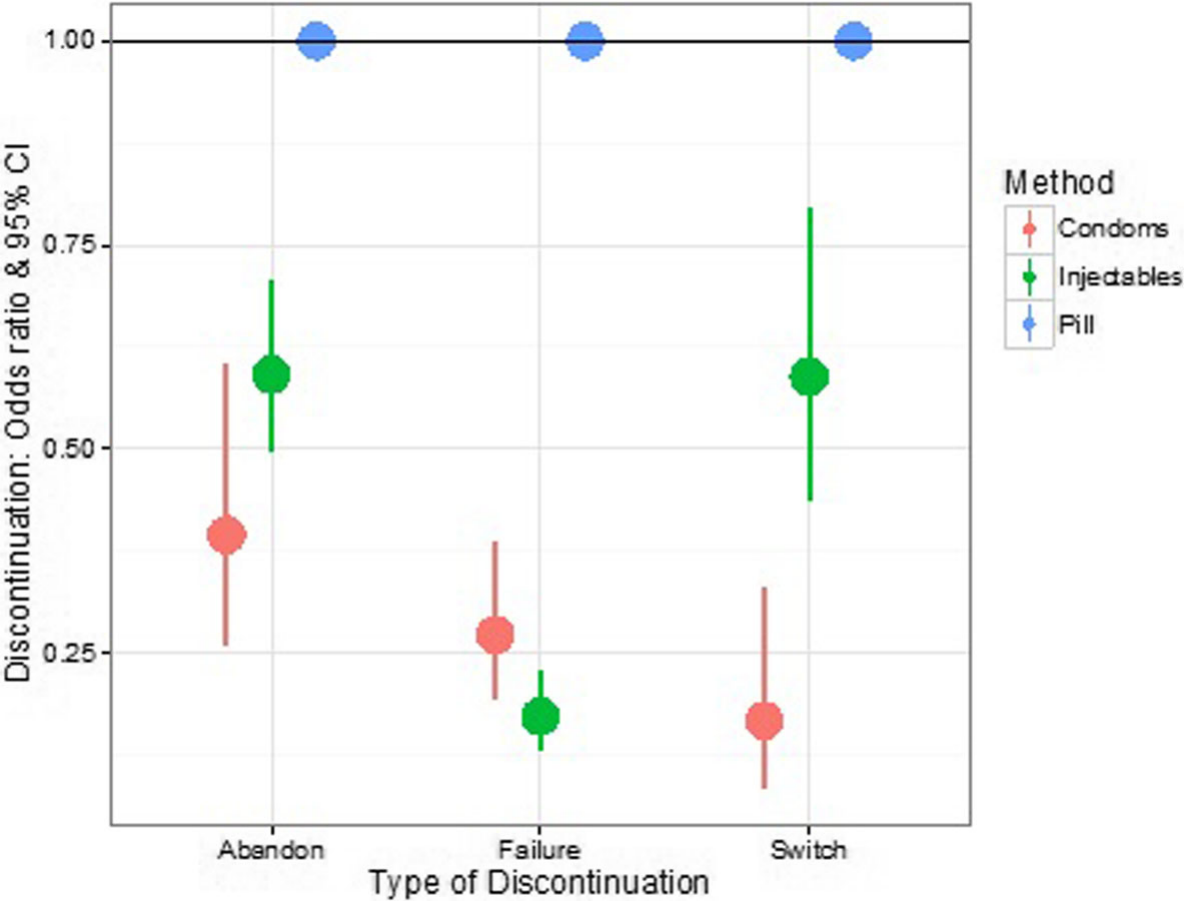
Predicted odd-ratios & 95% confidence intervals for contraceptive discontinuation as a function of method type. The analysis focuses on short-acting contraceptives only and the reference category (horizontal line) corresponds to the use of oral contraceptives. The risk of all three forms of discontinuation is higher for oral contraceptives. This figure is based on the results of a multilevel multiprocess model using the calendar data collected in the 2011 Ethiopian Demographic and Health Survey

The results show that the risks of all three types of discontinuation are the highest for oral contraceptives (Fig. 4): (i) The risk of abandonment is higher for the use of OC, compared to injectables (41% higher; OR = 0.59; 95CI [0.49; 0.70]) and condoms (61% higher; OR = 0.39; 95CI [0.26; 0.60]) (Table 4); (ii) The risk of failure is also higher for the use of OC, compared to injectables (83% higher; OR = 0.17; 95CI [0.13; 0.23]) and condoms (73% higher; OR = 0.27; 95CI [0.14; 0.56]) (Table 5); (iii) The risk of switch, a form of discontinuation generally considered to be a marker of women’s ability to choose between methods and a phenomenon that can reduce unmet needs, is the highest for oral contraceptives and the lowest for condoms (Table 6).

**Table 4:** Estimated coefficients and standard errors for hazards models of contraceptive abandonment in 2011. The results for a standard model and a multiprocess model, modelling method choice and discontinuation conjointly, are compared. In the multiprocess model, IUD/Implants are not considered due to the limitations of the aML software and only the results of the hazard model part are shown

Abandon 2011	Standard model		Multiprocess model	
Variable	Coefficient	SE	Coefficient	SE
Constant	*2.59*		*−0.960*	
Duration (month)				
0–12	*−0.723*		*−0.723*	
12–24	*0.560*		*0.560*	
24+	*0.020*		*0.020*	
Method				
Pill	0		0	
IUD/Implants	−2.801***	**0.305**	–	–
Injectables	−1.512***	**0.270**	−0.526***	**0.091**
Condoms	−0.069	0.144	−0.929***	**0.217**
Education				
No Education	0		0	
Primary	−0.559	0.337	−0.071	0.075
Secondary+	0.079	0.121	−0.127	0.093
Age at start				
< 25 years	0		0	
25–34 years	0.044	0.149	−0.460***	**0.100**
35–49 years	−0.148	0.172	−0.947***	**0.101**
Wealth				
Low	0		0	
Medium	−0.498**	**0.181**	−0.360***	**0.111**
High	−0.186	0.198	−0.539***	**0.102**
Area				
Urban	0		0	
Rural	−0.334	0.198	0.0007	0.081
Religion				
Orthodox	0		0	
Muslims	0.166	0.136	0.175**	**0.076**
Others	0.226	0.116	−0.225	0.442
Women level effects				
Sigma	0.30	0.24	0.406***	**0.106**
Rho (abandon/injectable)	***–***	***–***	0.215**	**0.093**
Rho (abandon/condom)	***–***	***–***	0.229	0.149
Rho (injectable/condom)	***–***	***–***	−0.092***	**0.035**

Italics depict fixed parameters, estimated previously using a simple model with no variables. Bold depicts significance: *P < 0.05, **P < 0.01, ***P < 0.001

**Table 5:** Estimated coefficients and standard errors for hazards models of contraceptive failure in 2011. The results for a standard model and a multiprocess model, modelling method choice and discontinuation conjointly, are compared. In the multiprocess model, IUD/Implants are not considered due to the limitations of the aML software and only the results of the hazard model part are shown

Failure 2011	Standard model		Multiprocess model	
Variable	Coefficient	SE	Coefficient	SE
Constant	*9.82*		*−4.10*	
Duration (month)				
0–12	*−0.156*		*−0.16*	
12–24	*0.228*		*0.228*	
24+	*−0.009*		*−0.009*	
Method				
Pill	0		0	
IUD/Implants	1.834***	**0.365**	–	–
Injectables	−2.959***	**0.532**	−1.763***	**0.144**
Condoms	−1.532***	**0.152**	−1.302***	**0.369**
Education				
No Education	0		0	
Primary	−0.971**	**0.347**	−0.168	0.141
Secondary+	−0.111	0.146	−0.357	0.179
Age at start				
< 25 years	0		0	
25–34 years	−0.254	0.188	−0.626**	**0.211**
35–49 years	−0.553**	**0.205**	−1.420***	**0.188**
Wealth				
Low	0		0	
Medium	−1.326***	**0.216**	0.169	0.219
High	0.258	0.261	−0.301	0.200
Area				
Rural	0		0	
Urban	−0.160	0.266	0.201	0.164
Religion				
Orthodox	0		0	
Muslim	0.277	0.177	0.408***	**0.136**
Other	0.477***	**0.135**	−0.116	3.181
Women level effect				
Sigma	0.43	0.62	0.489***	**0.133**
Rho (failure/injectable)	–	–	0.142	0.110
Rho (failure/condom)	–	–	0.170	0.155
Rho (injectable/condom)	–	–	−0.097***	**0.035**

Italics depict fixed parameters, estimated previously using a simple model with no variables. Bold depicts significance: *P < 0.05, **P < 0.01, ***P < 0.001

**Table 6:** Estimated coefficients and standard errors for hazards models of contraceptive switch in 2011. The results for a standard model and a multiprocess model, modelling method choice and discontinuation conjointly, are compared. In the multiprocess model, IUD/Implants are not considered due to the limitations of the aML software and only the results of the hazard model part are shown

Switch 2011	Standard model		Multiprocess model	
Variable	Coefficient	SE	Coefficient	SE
Constant	*9.81*		*−6.22*	
Duration (month)				
0–12	*−0.189*		*−0.19*	
12–24	*0.255*		*0.25*	
24+	*0.011*		*0.01*	
Method				
Pill	0		0	
IUD/Implants	0.399	0.412	–	–
Injectables	−0.436	0.249	−0.530***	**0.154**
Condoms	−0.646***	**0.155**	−1.792***	**0.349**
Education				
No Education	0		0	
Primary	−0.670	0.346	−0.118	0.186
Secondary+	−0.072	0.166	0.440**	**0.198**
Age at start				
< 25 years	0		0	
25–34 years	0.356	0.178	−0.256	0.230
35–49 years	−0.379	0.243	−0.517**	**0.235**
Wealth				
Low	0		0	
Medium	−0.642**	**0.251**	−0.041	0.280
High	−0.016	0.311	0.336	0.229
Area				
Urban	0		0	
Rural	0356	0.305	0.156	0.177
Religion				
Orthodox	0		0	
Muslim	0.235	0.162	0.029	0.170
Other	0.047	0.152	0.086	0.123
Woman level effects				
Sigma	1.029**	**0.39**	0.938***	**0.095**
Rho (switch/injectable)	–	–	0.083	0.079
Rho (switch/condom)	–	–	0.167	0.114
Rho (injectable/condom)	–	–	−0.103***	**0.035**

Italics depict fixed parameters, estimated previously using a simple model with no variables. Bold depicts significance: *P < 0.05, **P < 0.01, ***P < 0.001

##### Risk of discontinuation by other socio-demographic variables

The risk of all three forms of discontinuation is higher for younger ages (<25 years, Tables 4-6). Wealth modulates the risk of contraceptive abandonment, which is 42% lower among the wealthiest compared to the low income class (OR = 0.58; 95CI [0.48, 0.71], Table 4). Religion modulates the risks of both abandonment and failure, which are higher among the muslims compared to the Christian orthodox. Area does not modulate the risk of any form of discontinuation.

### Qualitative findings on the barriers to contraceptive continuation

The quantitative results provide a picture of contraceptive discontinuation in Ethiopia and its determinants. However they are unable to further explain the reasons and motivations for contraceptive use or discontinuation. Thus, although not directly comparable, the qualitative findings presented below aim to provide a more detailed understanding of the barriers to contraceptive continuation in Ethiopia.

#### Side-effects

Focus group discussion participants commonly cited that the major reason for discontinuation of hormonal contraceptives is the ‘excessive bleeding that women encounter at the beginning or after sometime of using the methods’. One mother of 3 from Arsi village with primary level education stated that “*As I started to take the injectable, I ended up with much bleeding [menstrual irregularities]*.” Bleeding is considered as a major side effect of contraceptive use that could lead to loss of life as a result of getting weaker due to losing too much blood from the body. In addition, the literal meaning of the term used to describe ‘excessive bleeding’, ‘dem bizat’, can be translated back into ‘too much blood’ in Amharic, is wrongly connoted with the effects of high blood pressure that can lead to an immediate death.

Other mentioned side effects as well as bleeding that women experienced that led them to discontinue hormonal methods include pain, dizziness, skin conditions and mood and appetite changes. For instance, although not included in the quantitative analysis, another hormonal method, the implant (commonly referred to in Ethiopia as ‘bars’) was cited by several women as being discontinued due to health concerns. For example, a married woman living in a small town in southern Ethiopian, with secondary education and two children, said that in the first few days after receiving the implant she felt restless and dizzy with severe and constant pain in her left arm and she was unable to hold things up in that hand. Having suffered from pain and psychological tension for nearly three months, she decided to have it removed. When asked about her main worry that led her to have it removed, she said that: “… *I do not want to lose my muscles. For me, it is better to have as many children as God let me, than getting paralyzed having gone against God’s will*”. Side effects reported by other women include “*Pills bring about irritation [scars] on the face*” (uneducated, 39, mother of 5, Arsi village) and “*the loop [IUD] brings about excessive bleeding and loss of appetite*” (primary school, 27, mother of 3, North Shewa small town). One health provider reported contraceptive users complaining of “*cases of excessive bleeding, increased blood pressure, headache, gastritis and others*” (Health Extension Worker, North Shewa small town).

Several participants, if they had not experienced side effects themselves, had heard rumours and reports of other women’s side effects which may make them more likely to abandon contraception themselves. Health concerns and rumours expressed by women in our study include: “*Taking pills causes stomach-ache due to the chemical reaction to our body cells*” (primary school achiever, 37, Sidama small town, mother of 3); “*Taking pills burns a woman’s face due to its chemical reaction in the body*” (uneducated women, 42, mother of 6, Arsi village); “*Pills can be concentrated in the womb and damage the woman’s reproductive organ*” (primary school achiever, 24, mother of 3, North Shewa village), “*A woman should not use injectables until she achieves her ideal family size as it causes secondary sterility*” (uneducated, 38, mother of 5, Arsi rural village). Health workers reported these rumours to have a significant effect on women’s use of contraception with one stating “*Some of our clients are just disappearing [fail to show up for contraceptive resupplies] without letting us knowing the reason for discontinuing. … We later on learnt that these women stop using the (family planning) method due to the widespread information about possible side effects of contraceptives among themselves which could be true or not*” (Nurse, Family Planning Provider, Arsi Village). Although not in all cases, many of these rumours appear to have a basis in the reports of what women believe they have actually experienced that then sometimes become amplified, distorted and wrongly explained through several occurrences of social transmission. Thus, many ‘misconceptions’ may have some grounding in real experience.

#### Poverty

The qualitative investigation added further detail to the quantitative finding that abandonment of contraception was predicted by wealth. There was a perception among women in the qualitative interviews that hormonal contraception is not well suited to the bodies of women in lower socioeconomic classes with a poor diet. For instance, one woman (primary school achiever, 32, mother of 3, North Shewa small town) stated that “*Only women having access to better diet [egg, meat, butter …] should take pills or injectables as it does not work for those having poor diet. … the tablets [pills]/ dose [injectables] need better diets to operate in the woman’s body*” and others report fears of hormonal contraceptives using up energy from their already small food intake and making them hungry or ill. Participants who underwent excessive bleeding reported abandoning contraception because they were told that their body was getting weaker and that they needed to take a balanced diet to replace the blood that they had already lost. Specifically, participants living in poverty often took the immediate action of stopping the method as the suggested solution was beyond their economic reach, and maintaining their health was their greatest concern. These findings suggest the possibility that side effects may be experienced to a greater extent or felt more severely in the lower socioeconomic statuses, leading to higher levels of abandonment.

#### The need for secrecy

Another barrier to continuation commonly cited in the interviews is the fear of a woman’s contraceptive use being found out by her husband. Many women reported that their husbands disapproved of contraception and associated it with infidelity. Therefore, if a woman experiences physiological side effects, it can compromise the secrecy of her contraceptive use which might force her to discontinue. The need to keep contraceptive use a secret from a male partner also hinders a woman’s ability to seek help or advice for side effects by contacting the family planning service providers. Indeed, women reported that to visit the nearby health facility to speak to a health care provider or get their resupply, they needed to have a convincing reason to avoid the suspicion of their husbands. As this was often not possible, women were more likely to abandon contraception. The account from one married rural woman with primary level education and mother of 4 children sums up well what women cited as to why their husbands were suspicious of the use of contraception.

“*I started using pills without the knowledge of my husband as he was not in favour of family planning services for his mental setting is to associate contraceptive use with sexual infidelity. He thinks that women are using contraceptives to enhance their chances of ‘cheating on their husbands’ as it prevents pregnancy that could occur outside marriage.*”

Raised suspicion from the husband for any reason and fear of him finding out, may cause a woman to have to abandon her use of contraception. It seems for many women there is no solution which does not completely eliminate this risk, as shown by the rest of this woman’s quote:

“*I decided to take oral pills to prevent pregnancy. As I had been taking the pills every night before going to bed (actually hiding it outside a bedroom) and drinking water to swallow it, he [husband] got suspicious of my act and began to question why I do that every night regularly. Hence, I decided to use another method that would not get him clue of my act to prevent pregnancy. Then I decided to switch to another method, the injectables initially. However, I found it difficult to do so as it requires visiting the nearby health post or health center at least every three months for which I could not come-up with ‘good reasons’ to do so regularly. My intention to use implants did not also work out as the scars on my arms could alert the suspicion of my husband. Whilst consulting my closest friend living in a nearby town and seeking advice from her, I learnt that an IUD is the safest as it can stay for years without necessarily going through resupply every time. Though I have successfully got the IUD inserted, I got to be worried about my husband’s complaints of “sexual pleasure” since recently. He repeatedly complained that he feels something strange in my body during sexual consummation, and asked if I did anything that is unknown to him. Having realized that his question was emanating from the discussion he had with his male friends that do not support family planning services, I decided to remove the IUD to save my marriage and maintain ‘peace’ in my marital life and the wellbeing of our children.*”

This fear of repercussion if their husbands were to find out about their contraceptive use was echoed throughout the discussions and a general disapproval and distrust from men of use of contraception was reported. However even among women themselves, there is some sense of association between use of contraception and infidelity with opinions being expressed such as: “*Women who are loyal to their husbands should not use condoms*” (primary school, 26, mother of 1, North Shewa village) and “*The idea of using a condom creates suspicion between the wife and the husband*” (secondary school, 33, mother of 5, Arsi small town). However even if a woman does not hold these associations and wishes to continue taking contraception to limit or space her births, it may be very hard for her to do so if she fears the consequences of her husband finding out she is taking it. This may in part explain the popularity of injectables, as along with the educational campaign held in the country in the late 1990s to make injectables popular, they afford secrecy by leaving no trace on the body and only need to be administered once every 3 months and thus require fewer excuses to visit the health centre.

## Discussion

Contraceptive discontinuation has been identified as a major challenge to tackling unmet needs of contraception and unwanted fertility in the developing world [7–9]. This paper aims to provide a better understanding of the reasons why women discontinue contraception in Ethiopia, a country where 1 in 5 women has an unmet need for contraception (DHS data) despite a nine-fold increase in the number of women who have ever used contraception. Complementing an analysis of the 2011 EDHS data with semi-structured interviews with Ethiopian women and health professionals in 2013, the results show that (1) overall 36 months rates of discontinuation have not decreased in the period 2005–2011 (from 49.4% in the 2005 EDHS (analysis not shown) to 56.5% in the 2011 EDHS), (2) discontinuation is significant and multifactorial, and mainly takes the form of contraceptive abandonment (2/3 of all discontinuation rates) as compared to switch and failure, (3) contraceptive abandonment is a function of method type, age, wealth and religion, but is independent from education, and (4) important barriers to continuation are (i) the experience of physiological side-effects associated with the use of hormonal contraceptives, (ii) the conflation of contraceptive use with the occurrence of marital infidelity, and (iii) poverty. Taken together, the results show that female level of education is not the main barrier to contraceptive continuation, and suggest a shift in perspective focusing on tackling male perceptions over women’s reproductive decision-making, greater targeted reproductive and sexual health education as well as evaluating the appropriateness of medical technology to the physiology of Ethiopian women.

### Contraceptive abandonment is the main form of discontinuation

The overall 36 months risk of discontinuation of modern contraception is just over 50%, and out of the three forms of contraceptive discontinuation studied, i.e. abandonment, failure, and method switch, abandonment accounts for 2/3 of all discontinuation rates. Contraceptive abandonment does not always lead to unwanted pregnancy, however, as there are many reasons why women might stop using contraceptives, e.g. desire for more children, no further needs, method-related reasons and health concerns/side-effects. In the 2005 Ethiopia DHS, Ali, Cleland and Shah found that no further need and the desire for another child accounted for just 15% and 33%, respectively, of the overall rate of contraceptive discontinuation [8]. This suggests that although abandonment is not always the results of method-related reasons and health concerns, those reasons do account for a significant part of the overall rate of discontinuation in Ethiopia. The 2011 DHS data does not allow quantifying the contribution of each type of reason to the overall abandonment rate as a reason for discontinuation was not included, but our analysis shows that the risk of abandonment is a function of the type of method used.

### Contraceptive abandonment, method type and the need for secrecy

Oral contraceptives (OC) are ca. 40% and 60% more likely to be abandoned than injectables and condoms, respectively. This picture differs from the results obtained in a previous analysis of 23 developing countries, where the 12 months probability to discontinue due to method-related reasons is found to be, on average, the highest for injectable contraceptives compared to OC or condoms [34]. Yet, substantial variation in the discontinuation rate of injectables was found between countries (from ca. 24% to 42%), overlapping with the range of discontinuation rates observed for oral contraceptives (from ca. <10% to >50%) [34]. A possibility for explaining why injectables are not more discontinued than OC is that although injectables and oral contraceptives are both associated with side-effects, injectables afford a benefit oral contraceptives do not. In the qualitative interviews, one determinant of discontinuation that emerged as critical in reducing women’s ability to continue taking contraception, switch method or seek advice, was the association of contraceptive use with infidelity and thus the need for keeping contraceptive use secret. Fear of husband’s suspicion of contraceptive use might go some way to explaining the popularity of injectables- a quarter of married women and a third of unmarried sexually active women use injectables (EDHS 2016) - as it leaves no trace on the body and, compared to OC, it only needs to be administered once every 3 months thus minimizing the number of visits to the health centre.

### The experience of physiological side-effects

The qualitative data indicate that women experience debilitating side-effects, which amongst others come in the form of headaches, nausea and excessive bleeding, which is interpreted as a threat to a woman’s health and her ability to conceive. Given a woman’s capacity to reproduce has traditionally been a critical asset in her marital union [35], the experience of side-effects further puts women at risk with their marriage. The findings are in line with a recent report from the UNFPA suggesting that the main reasons cited for not using “family planning worldwide are the fear of side effects and health concerns” [36]. While the literature on side-effects is generally unclear with regards to the distinction between “real” side-effects and unsubstantiated rumours [37, 38], our findings indicate that the fear of many types of side-effects is likely to originate in the experience of users. Why women experience physiological side-effects might result from a mismatch [13] between the dose of hormones found in the contraceptives used in Ethiopia and the physiology of Ethiopian women. Although a clear general pattern is difficult to draw, especially when taking within populations comparisons [20], levels of ovarian steroids are often found to be higher for women living in rich environments and lower for women living in ecologies characterized by high physical workload [39] and nutritional stress [20, 40]. For instance, as compared with women living in the US, salivary progesterone levels are significantly lower for women living in Zaire [41], Nepal [42] and Poland [39]. If further analysis reveals that side-effects result from a mismatch between local hormonal levels and those present in the contraceptives obtained from international donors, then a focus on tackling the cause of side-effects through developing new contraceptives adapted to the local reproductive ecology may have drastic consequences for decreasing unmet needs for contraception.

### Education and contraceptive discontinuation

The results show that less educated women are not more or less likely to abandon contraceptive use as compared to highly educated women. This result is not new and has been previously observed in a study on oral contraceptive discontinuation in 19 developing countries [7]. In addition, a review of 6 developing countries found that pill continuation was overall not correlated with a woman’s level of education and concluded that counselling for uneducated women was not in need of radical review [43]. One can argue that education level might not capture a woman’s level of reproductive health knowledge, however. Yet, it is likely that education level reflects, at least partly, biomedical understanding of reproduction as more educated women are found to be more likely to switch methods. Thus, if formal education alone does not influence rates of contraceptive abandonment and failure, it is relevant for understanding the rate of contraceptive switching [44, 45].

With regards to tackling contraceptive abandonment, more specific reproductive health education and counselling may prove more effective than formal education. However, in many family planning programmes, policy on counselling women includes reassuring them about side effects and encouraging them to continue taking the contraceptive. For instance, recent reports suggest that to reduce high rates of discontinuation, women should be “forewarned about side-effects and reassured about health concerns” [8] and another report suggests “dispelling misconceptions and counselling women who experience amenorrhea” [9]. While information is undoubtedly mandatory, it is not sufficient. In a country such as Ethiopia where contraception is frequently used for spacing births [46, 47], it is no surprise that the experience of physiological side-effects that weaken the body is a major barrier to continuation. It has been shown that in sub-Saharan Africa, contraceptives may be used to heal and maintain strength in the face of repeated pregnancies and reproductive mishaps, with the decline in bodily resources being seen as the most significant barrier to achieving high fertility [48]. It follows that the recommended best practice in Ethiopia in the face of side-effects - “If a client experiences common side-effects […], you should advise the woman to keep taking her pills” [49] – may be beyond the reach of most women as their experience is too debilitating [19]. The qualitative findings show that even health workers believed the recommended counselling did not give the experience of side-effects enough gravity, or even credence:

*“The service provision manual provided to us by the Federal Ministry of Health of the country [Ethiopia] states that most of the complaints raised by the clients are based on ‘rumours’. However, we have really noticed that there are some kinds of change on the bodies and faces of our clients over time. … I can’t totally rule out the possible side-effects of contraceptives that we are providing at the moment”. (Nurse, Family Planning provider at Arsi, Central Ethiopia)*

### Implications

#### Engaging men

The qualitative results point to the importance of men’s opinion of family planning and the need for informative, confidential service provision as women’s fear of men’s discovery of their use of contraception is a significant barrier to them being able to meet their need for contraception. This has implications for increasing involvement of men in Ethiopia’s family planning programmes and report on reducing discontinuation suggests that engaging male partners by “enhancing couple communication about method characteristics can be effective in supporting continued use, particularly in the postpartum period” [9]. Interventions targeting male leaders, husbands and men and encouraging greater acceptance of use of contraception may indeed allow women to seek counselling about any side effects and get resupplies closer to home.

#### Education of service providers

The online resource available for family planning counselling on oral contraceptive pills in Ethiopia [49] begins with listing common “myths”: “Women who stop taking the pill may not be able to get pregnant; they become infertile; the pill causes cancer; Oral pills build up in a woman’s body”. While misconception is perceived as the most pressing issue, it is only a few pages later that the most common side-effects, i.e. changes in menstrual bleeding patterns (irregular periods, spotting, amenorrhoea), headache, nausea, sore breasts, mood changes and sometimes high blood pressure, are covered. In this context, changing the order of topics covered in the counselling material may help initiate a shift away from conceptualising side effects as rumours or minor problems to acknowledging real problematic and sometimes intolerable experiences. Providing service providers with the training necessary for distinguishing between substantiated concerns (i.e. irregular bleeding) and myths would enable them evaluating when to tackle misconception and when to counsel switch to a different method and/or other solutions. Note however that many of the interventions that may help alleviate some of the worst side effects, such as painkillers or iron supplements to replace that lost through irregular bleeding, are out of the reach of many women in Ethiopia.

#### Tackling the root cause of side-effects

A commonly cited reason for the abandonment of hormonal contraceptives (pill, injectables, IUD) is the experience of side effects that can both jeopardise the maintenance of secrecy and be too debilitating to enable the continuation of contraceptive use. If the experience of side effects is found to be representative of Ethiopian women, especially those living in poverty, finding out the root cause of those side-effects may go a long way in tackling discontinuation and decreasing rates of unwanted pregnancy. Discontinuation due to side-effects is widespread in low and middle income countries [34], and it has been argued that debilitating symptoms may arise due to a mismatch between the dosage of hormonal contraceptives and endogenous levels of hormones in non-Westernised populations [13, 50]. Such possibility remains to be demonstrated in Ethiopia.

If the mismatch hypothesis is revealed to be pertinent for explaining the occurrence of physiological side-effects among Ethiopian women, a potential solution could be to provide users with a lower-dose injectable, for instance the Sayana Press, a self-administrated injectable that is now licensed in many countries. However, this product was tested in U.S. and Singaporean women, in samples with a mean BMI of 28.7 and 22.4 respectively [51], and comes with special warnings about high risk of menstrual irregularities and loss of bone mineral density. Further, the US sample was comprised of 41% overweight women (with BMIs >30) who are likely to have physiologies far removed from that of a rural Ethiopian woman living in poverty. Thus there is a need of trialling contraceptives on populations of women living in different ecologies before determining their acceptability.

### Limitations

We have used both semi-structured interviews and DHS data but the information collected in both cases is not directly comparable. Indeed, the quantitative data have been collected 2-years before the interviews were conducted, and the sample of women interviewed represents a small sub-set of the nationally representative DHS data. For instance, only ever-married women were included in the qualitative analysis due to the cultural unacceptability of discussing sensitive issues related to sexual behaviour before marriage. In addition, due to a small sample size, the qualitative findings cannot be interpreted as representative of the socio-economic status or education level. It follows that not all reasons for discontinuation have possibly been voiced. This combined with the absence of data on the reasons for discontinuation in the 2011 EDHS data means that the quantification of the contribution of each of the possible reasons to discontinuation rates is beyond the scope of this paper. Yet, the findings that (i) at the national level, discontinuation depends on the type of contraceptive method and (ii) among married women in need of contraception, the experience of physiological and social side effects promotes contraceptive discontinuation, together point towards method related issues as a root cause for discontinuation.

## Conclusions

In line with recent reports on improving family planning programs worldwide [9, 36], we conclude that for Ethiopia, a strategy prioritizing the tackling of discontinuation due to side-effects over the increasing of the number of new clients may have drastic consequences for decreasing unmet needs for family planning. Further, to tackle discontinuation due to side-effects, we argue that dispelling misconceptions through educating women is not addressing the root causes of discontinuation, and that priority should be given to (i) engaging men, (ii) acknowledging the importance of real side effects in counselling material and (iii) evaluating the suitability of currently used contraceptives to the physiology of Ethiopian women.

## Abbreviations

95CI: 95% Confidence Interval
BMI: Body Mass Index
DHS: Demographic and Health Survey
EDHS: Ethiopian Demographic and Health Survey
FGD: Focus Group Discussion
FMOH: Federal Ministry of Health
FP: Family Planning
IAA: Independence of Irrelevant Alternatives
IUD: Intra Uterine Device
OC: Oral Contraceptives
OR: Odd-Ratio
SNNP: Southern Nations, Nationalities, and Peoples’ Region
TFR: Total Fertility Rate
U.S: United States
UNFPA: United Nations Population Fund (United Nations Fund for Population Activities)
